# Multimodal MRI of grey matter, white matter, and functional connectivity in cognitively healthy mutation carriers at risk for frontotemporal dementia and Alzheimer's disease

**DOI:** 10.1186/s12883-019-1567-0

**Published:** 2019-12-27

**Authors:** Rogier A. Feis, Mark J. R. J. Bouts, Elise G. P. Dopper, Nicola Filippini, Verena Heise, Aaron J. Trachtenberg, John C. van Swieten, Mark A. van Buchem, Jeroen van der Grond, Clare E. Mackay, Serge A. R. B. Rombouts

**Affiliations:** 1 Department of Radiology, Leiden University Medical Centre, Leiden, The Netherlands; 2 FMRIB, Oxford Centre for Functional Magnetic Resonance Imaging of the Brain, Nuffield Department of Clinical Neurosciences, University of Oxford, Oxford, UK; 3 LIBC, Leiden Institute for Brain and Cognition, Leiden, The Netherlands; 4 Institute of Psychology, Leiden University, Leiden, The Netherlands; 5 Department of Neurology, Erasmus Medical Centre, Rotterdam, The Netherlands; 6 Department of Psychiatry, University of Oxford, Oxford, UK

**Keywords:** Microtubule-associated protein tau, progranulin, Apolipoprotein E4, Voxel-based morphometry (VBM), diffusion tensor imaging (DTI), Tract-based spatial statistics (TBSS), functional connectivity, Dual Regression Analysis, Frontotemporal dementia, Alzheimer’s disease

## Abstract

**Background:**

Frontotemporal dementia (FTD) and Alzheimer’s disease (AD) are associated with divergent differences in grey matter volume, white matter diffusion, and functional connectivity. However, it is unknown at what disease stage these differences emerge. Here, we investigate whether divergent differences in grey matter volume, white matter diffusion, and functional connectivity are already apparent between cognitively healthy carriers of pathogenic FTD mutations, and cognitively healthy carriers at increased AD risk.

**Methods:**

We acquired multimodal magnetic resonance imaging (MRI) brain scans in cognitively healthy subjects with (*n*=39) and without (*n*=36) *microtubule-associated protein Tau* (*MAPT*) or *progranulin* (*GRN*) mutations, and with (*n*=37) and without (*n*=38) *apolipoprotein E ε4* (*APOE4*) allele. We evaluated grey matter volume using voxel-based morphometry, white matter diffusion using tract-based spatial statistics (TBSS), and region-to-network functional connectivity using dual regression in the default mode network and salience network. We tested for differences between the respective carriers and controls, as well as for divergence of those differences. For the divergence contrast, we additionally performed region-of-interest TBSS analyses in known areas of white matter diffusion differences between FTD and AD (i.e., uncinate fasciculus, forceps minor, and anterior thalamic radiation).

**Results:**

*MAPT/GRN* carriers did not differ from controls in any modality. *APOE4* carriers had lower fractional anisotropy than controls in the callosal splenium and right inferior fronto-occipital fasciculus, but did not show grey matter volume or functional connectivity differences. We found no divergent differences between both carrier-control contrasts in any modality, even in region-of-interest analyses.

**Conclusions:**

Concluding, we could not find differences suggestive of divergent pathways of underlying FTD and AD pathology in asymptomatic risk mutation carriers. Future studies should focus on asymptomatic mutation carriers that are closer to symptom onset to capture the first specific signs that may differentiate between FTD and AD.

## Background

Frontotemporal dementia (FTD) and Alzheimer’s disease (AD) are two of the most common causes of dementia [1–4]. In addition to distinct clinical features [5–9], FTD and AD demonstrate different patterns of functional and structural neurodegeneration on magnetic resonance imaging (MRI) [10–17]. Atrophy is more pronounced in FTD than in AD in frontotemporal areas such as the anterior cingulate cortex, fronto-insula, and inferior frontal cortex [10, 13, 15]. Conversely, AD patients have more atrophy in the occipital gyrus and precuneus than FTD patients [13]. In terms of white matter diffusion tensor imaging (DTI) alterations, FTD patients have reduced fractional anisotropy (FA) and increased radial diffusivity (RD) compared to AD patients in the uncinate fasciculi, forceps minor, and anterior thalamic radiation, whereas AD patients do not show FA decreases or RD increases compared to FTD patients [11, 13–16]. Furthermore, functional connectivity is inversely affected in FTD and AD. In FTD patients, functional connectivity with the salience network is disrupted, while functional connectivity with the default mode network is increased. Vice versa, functional connectivity with the default mode network is disrupted in AD patients, while functional connectivity with the salience network is increased [12, 17].

Despite these different patterns of neurodegeneration, the differentiation between FTD and AD is often demanding when patients first present in the memory clinic. For example, FTD patients may first present with memory deficits [18, 19], and as such may be misdiagnosed as AD patients. Conversely, AD patients may be misdiagnosed as FTD patients due to the presentation of behavioural symptoms [20]. Indeed, 13% of initial FTD diagnoses were corrected to AD after two years follow-up [21], while 10–30% of clinical FTD patients were found to have AD pathology upon autopsy [22–24].The current criteria for behavioural variant FTD (bvFTD) [5], and language FTD variants [6] lack specificity to distinguish early-stage FTD patients from early-stage AD patients [7]. This diagnostic problem delays effective disease management [21, 25–27], and frustrates the development of new treatments. Considering that the potential of disease-modifying drugs is highest in the stage before atrophy occurs, the identification of early-stage dementia patients is crucial for patient selection in clinical trials [28].

To assess whether FTD- and AD-related pathological changes are present even before symptom onset, carriers of FTD and AD risk mutations have been studied using structural, diffusion-weighted, and functional MRI (fMRI). For example, mutations in *microtubule-associated protein Tau* (*MAPT*), *progranulin* (*GRN*), and repeat expansions in *chromosome 9 open reading frame 72* (*C9orf72*) are known causes of genetic FTD. Presymptomatic carriers of these mutations have therefore been regularly used to investigate early-stage FTD-related pathology [29–33]. Similarly, mutations in *presenilin 1*, *presenilin 2*, and *amyloid precursor protein* are known causes of genetic AD. However, due to its higher prevalence, *apolipoprotein E ε4* (*APOE4*), the strongest risk factor for sporadic AD, has been more extensively used to study early-stage AD-related pathology [34–43].

Contrary to findings in clinical FTD and AD [11, 13–16], differences in diffusion metrics associated with asymptomatic *APOE4* [39, 44–51] are more widespread than diffusion differences associated with asymptomatic *MAPT/GRN* mutation carriers [32, 33]. Functional connectivity differences have also been shown in these asymptomatic groups [32, 41]. However, a comparison between these presymptomatic patterns of change in risk mutation carriers for FTD and AD is lacking, even though early-stage differences between these dementias may aid early differential diagnosis.

To this end, we investigated multimodal MRI in asymptomatic subjects at risk for FTD and AD. First, we aimed to replicate early carrier-control differences found between *MAPT*/*GRN* mutation carriers and controls, and between *APOE4* carriers and controls, respectively, by assessing whole-brain grey matter volume, white matter DTI measures, and functional connectivity in the default mode network and salience network. Secondly, we investigated whether *MAPT/GRN* carrier-control differences diverged from *APOE4* carrier-control differences, similar to FTD-AD differences. For the latter analysis, we additionally evaluated a priori selected white matter tracts known to be affected more strongly in FTD than AD (i.e., uncinate fasciculus, forceps minor, and anterior thalamic radiation). We hypothesised that the differences in grey matter volumes, DTI measures, and functional connectivity seen in FTD and AD patients [10–17] may also be present to a smaller extent before symptom-onset in risk mutation carriers.

## Materials and methods

### Participants

Subjects were included retrospectively from studies carried out at the Leiden University Medical Centre (LUMC), The Netherlands, and at the Functional Magnetic Resonance Imaging of the Brain Centre (FMRIB), Oxford, UK.

The Dutch sample included 39 *MAPT/GRN* mutation carriers (11 *MAPT*, 28 *GRN*) and 36 controls, recruited from a pool of 160 healthy first-degree relatives of FTD patients with either *MAPT* or *GRN* mutation [32]. Participants were considered asymptomatic in the absence of (1) behavioural, cognitive, or neuropsychiatric change reported by the participant or knowledgeable informant, (2) cognitive disorders on neuropsychiatric tests, (3) motor neuron disease signs on neurologic examination, and (4) other FTD [5, 6] or amyotrophic lateral sclerosis [52] criteria. Asymptomatic non-carriers from these families and the general population were assumed to have equal risk of developing dementia. *MAPT/GRN* mutation carriers and controls were not tested for *APOE4* alleles.

Data from 37 *APOE4* carriers (30 *apolipoprotein E ε3/ε4* heterozygotes, 7 *apolipoprotein E ε4/ε4* homozygotes) and 38 controls (all *apolipoprotein E ε3/ε3* homozygotes) were collected in Oxford from the general population in Oxfordshire and were selected to match the Dutch sample in terms of age and gender. Due to the limited sample size, it was not possible to match the groups' education level. Middle-aged and elderly *APOE4* carriers and controls underwent a pre-screening cognitive test (Addenbrooke's Cognitive Examination-revised version [39, 40]) to assure asymptomatic status. *APOE4* carriers and controls were not tested for *MAPT/GRN* mutations.

In both cohorts, participants were between 21 and 70 years old. A priori exclusion criteria included MRI contraindications, head injury, current or past neurologic or psychiatric disorders, (history of) substance abuse including alcohol, corticosteroid therapy, type I diabetes therapy, and memory complaints.

The study was conducted in accordance with regional regulations and the Declaration of Helsinki. Written informed consent was received from all participants, and ethical approval for data acquisition was provided by the Medical Ethical Committees in Rotterdam and Leiden for *MAPT/GRN* data, and the National Research Ethics Service Committee South Central – Oxford C for *APOE4* data. For further details regarding the recruitment protocols, see Dopper et al. (2014) [32] for the Dutch sample and Filippini et al. (2011) [40] for the English sample.

### Image acquisition

MRI data were acquired with a Philips 3 T Achieva MRI scanner using an 8-channel SENSE head coil (*MAPT/GRN* mutation carriers and controls) or on a Siemens 3 T Trio scanner with a 12-channel head coil (*APOE4* carriers and controls). T1-weighted data were acquired with TR=9.8 ms, TE=4.6 ms, flip angle=8°, 140 axial slices, and voxel size is 0.88 x 0.88 x 1.20 mm for *MAPT/GRN* mutation carriers and controls, and using a magnetisation-prepared rapid gradient echo sequence (MPRAGE; TR=2040 ms, TE=4.7 ms, flip angle=8°, 192 axial slices, voxel size is 1 x 1 x 1 mm) in *APOE4* carriers and controls. Diffusion-weighted images were acquired in 62 directions with TR=8250-9300 ms, TE=80-94 ms, b-value=1000 s/mm^2^, flip angle=90°, 65-70 axial slices, and voxel size is 2 x 2 x 2 mm. For the resting-state functional MRI (rs-fMRI) scan, subjects were instructed to remain awake and keep their eyes closed (*MAPT/GRN* mutation carriers and controls) or open (*APOE4* carriers and controls), and to think of nothing in particular. We acquired 180-200 volumes with TR=2000-2200 ms, TE=28-30 ms, flip angle=80-89°, and voxel size is 2.75 x 2.75 x 2.75 mm + 10% interslice gap or 3 x 3 x 3.5 mm.

### Image analysis

FMRIB’s Software Library (FSL, http://www.fmrib.ox.ac.uk/fsl) tools were used for all data analyses [53].

#### Grey matter volume analyses

Whole-brain voxel-wise structural analysis was carried out with FSL-VBM [54], an optimised voxel-based morphometry protocol [55] using FSL tools [56]. First, we performed brain extraction and grey matter segmentation, and registered images to the MNI-152 standard space using linear (FLIRT) and non-linear registration (FNIRT [57]). The resulting images were averaged and flipped along the x-axis to create a study-specific grey matter template. Native grey matter images were then re-registered to this template, modulated using the field-warp Jacobian, and smoothed using an isotropic Gaussian kernel with a sigma of 2.5 mm (~ 6 mm full width at half maximum).

#### Diffusion tensor imaging

Diffusion-weighted imaging scans were processed using FMRIB’s Diffusion Toolbox (FDT, http://www.fmrib.ox.ac.uk/fsl/fdt). First, we aligned raw diffusion weighted images to the b0-volume using “eddy correct” to correct for movement and eddy currents. Next, we fitted the diffusion tensor model to the images at each voxel to create modality-specific images for fractional anisotropy (FA), mean diffusivity (MD), axial diffusivity (AxD), and radial diffusivity (RD). For voxel-wise analysis of these images, we used tract-based spatial statistics [58]. After brain extraction, subjects’ individual FA images were transformed to standard space using FNIRT. A mean FA image then was created and thinned to generate a whole-brain mean FA skeleton, representing the centres of all white matter tracts common to all subjects. Individual aligned FA images were projected onto this skeleton for group analysis. Similar analyses were performed on MD, AxD, and RD maps using the spatial transformation parameters that were estimated in the FA analysis. For our region-of-interest analyses, we masked the whole-brain skeleton with the combined masks of the uncinate fasciculi, forceps minor, and the bilateral anterior thalamic radiations, which have been shown to differ between FTD and AD patients in terms of DTI metrics [11, 13–16].

#### Resting-state functional MRI

Pre-statistical processing of resting-state data consisted of motion correction [59], brain extraction, spatial smoothing using a Gaussian kernel of 6 mm full width at half maximum, 4D grand-mean scaling and high-pass temporal filtering corresponding to a period of 150s (~ 0.007 Hz). Registration to MNI-152 standard space was carried out in two steps. We registered echo-planar images onto their respective T1-weighted structural images using FLIRT and Boundary-Based Registration [59–61]. Next, we used FNIRT to align T1-weighted structural images to MNI-152 standard space, and concatenated the resulting registration matrices to register echo-planar images directly to standard space. Next, we performed individual Independent Component Analysis (ICA) and voxel-wise intensity normalisation (i.e., by dividing all voxels by their time series’ mean values and multiplying by 10,000).

We used FMRIB’s ICA-based X-noiseifier (FIX [62–64]) to clean up noise components and reduce rs-fMRI scan site bias. For a detailed description and validation of FIX as a multicentre bias reduction method, see Feis et al. (2015) [64]. In short, we classified the individual ICA components of a subset of the subjects as signal, noise or unknown, trained the FIX classifier, and used a leave-one-out test to control the algorithm’s quality. All subjects’ data were then classified using the optimal threshold (i.e., 20 – true-positive rate 95.1%, true-negative rate 91.4%) and structured noise components were removed.

**Fig. 1:**
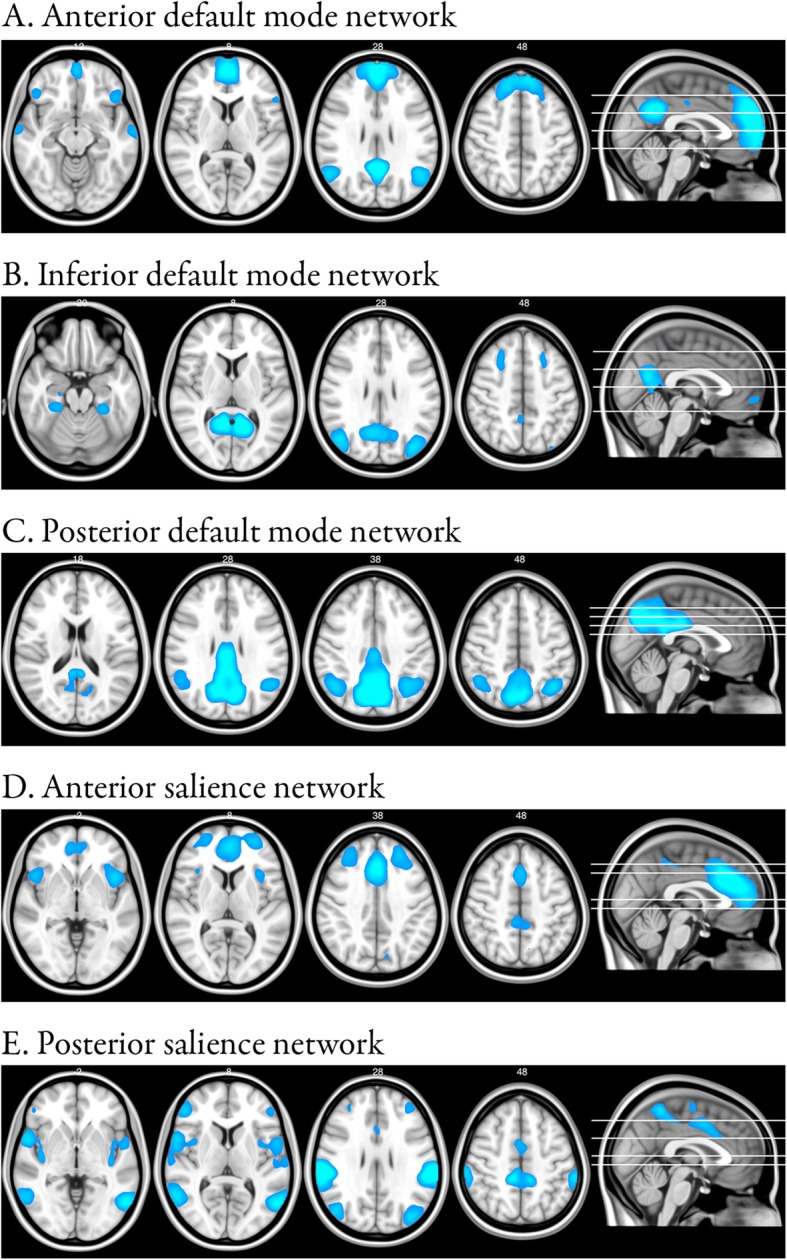
Resting-state networks. Maps illustrate the most informative slices of resting-state networks of interest that featured known default mode network and salience network regions and that were used for statistical testing after dual regression

After processing and application of FIX, rs-fMRI data were temporally concatenated and decomposed into 25 components using FSL’s group-level ICA tool [65–67], in order to identify large-scale patterns of functional connectivity. The resulting group-level ICA spatial maps were compared to previously described resting-state networks [67–71], and we selected default mode network components and salience network components for dual regression analyses. The default mode network is disrupted in AD, and enhanced in FTD, while the salience network is disrupted in FTD and enhanced in AD [12, 17]. Components that included the precuneus, posterior cingulate cortex, angular gyrus, medial pre-frontal cortex and hippocampus were regarded as parts of the default mode network. Components featuring the anterior cingulate cortex, supplementary motor area and insula were considered linked to the salience network. We found three networks resembling the default mode network (e.g., the anterior, inferior and posterior default mode network, Fig. 1a-c) and two networks resembling the salience network (e.g., the anterior and posterior salience network, Fig. 1d-e). For these five resulting resting-state networks of interest, we performed dual regression to identify the subject-specific spatial maps corresponding to the resting-state networks of interest [37, 72]. First, the spatial maps derived from group-level ICA were used as a spatial regressor in each subjects’ rs-fMRI data to obtain subject-specific time series describing the temporal dynamics for each component (Additional file 1: Figure S1, step 1). Next, the time series found by spatial regression were used as a temporal regressor to find the voxels associated with those time series for each subject (Additional file 1: Figure S1, step 2). As such, we used the group-level ICA networks of interest to obtain subject-specific spatial maps that allow for voxel-wise comparison. Statistical analysis of region-to-network functional connectivity group differences was then carried out by testing for the functional connectivity between the five resting-state networks of interest and all other grey matter voxels.

### Statistical analysis

Statistical analysis of grey matter volume, DTI features, and rs-fMRI data was performed using general linear models, including age and education as confound regressors. Additionally, we added a voxel-wise covariate for grey matter volume to the functional connectivity analyses. We tested for differences between, respectively, *MAPT/GRN* mutation carriers and controls, and differences between *APOE4* carriers and controls. Additionally, we tested for the differences between these respective carrier-control contrasts to evaluate whether these gene mutations have divergent effects on the brain in cognitively healthy carriers that might reflect early substrates of FTD or AD pathology. Since possible centre effects are equivalent for carriers and controls at each site, these effects cancel out when we compared the carrier-control effect at one site to the carrier-control effect at the other site. Consequently, unknown confounding factors such as scanner and population differences should have minimal influence on our results.

Pooling *MAPT* and *GRN* mutation carriers, and *APOE4* heterozygotes and homozygotes in our carrier samples may have increased heterogeneity in our groups. To account for this possibility, we performed additional analyses with covariates encoding the difference between *MAPT* and *GRN* mutations, and between *APOE4* hetero- and homozygosity.

Voxel-wise application of these general linear models to the data was performed using FSL randomise, a permutation-based non-parametric test (5,000 permutations). We set the family-wise error rate at 5% across space by using threshold-free cluster enhancement [73] in all analyses. The alpha level required for statistical significance was set at 0.025 for all imaging analyses, which corresponds to an alpha level of 0.05 in a two-sided *t*-test, since randomise performs the permutation equivalent of a one-sided *t*-test. Minimal cluster size for significant results was set at 10 voxels.

SPSS version 24 (SPSS, Chicago, IL) was used for statistics performed on non-imaging (demographic) variables. Analysis of variance (ANOVA) tests were performed on normally distributed continuous variables (age and education) and included Bonferroni post-hoc tests. A *χ*^2^ test was performed for gender. The alpha level required for statistical significance was set at 0.05.

## Results

### Demographics

Demographic data for all groups are shown in Table 1. Age and gender did not differ between groups. Bonferroni post-hoc tests revealed significantly lower education level in years for *MAPT/GRN* mutation carriers than *APOE4* controls (*p*=0.001), for *MAPT/GRN* controls than *APOE4* controls (*p*<0.001), and for *MAPT/GRN* controls than *APOE4* carriers (*p*=0.001).

**Table 1:** Participant demographics

	*MAPT/GRN*		*APOE4*		*P*-value
	Carriers (*n*=39)^a^	Controls (*n*=36)	Carriers (*n*=37)^b^	Controls (*n*=38)	
Age, y^c^	50.5 (10.0)	49.8 (11.3)	48.6 (10.3)	50.5 (10.5)	0.855
Gender, % Female	23 (59%)	18 (50%)	20 (54%)	20 (53%)	0.885
Education, y^c^	14.0 (2.5)	12.6 (2.9)	15.5 (3.7)	16.8 (3.2)	< 0.001*

*APOE4, Apolipoprotein E ε4* carriers; *MAPT/GRN, Microtubule-associated protein Tau / progranulin* carriers.

^a^11 *MAPT* 28 *GRN*.

^b^30 heterozygotes, 7 homozygotes.

^c^Values denote mean (SD); education values were missing for three MAPT/GRN carriers and two *MAPT/GRN* controls.

*statistically significant at *p* < 0.05.

### Grey matter volume

We found no grey matter volume differences in *MAPT/GRN* mutation carriers compared to controls or *APOE4* carriers and compared to controls, nor were there differences between both contrasts.

### White matter diffusion

**Fig. 2:**
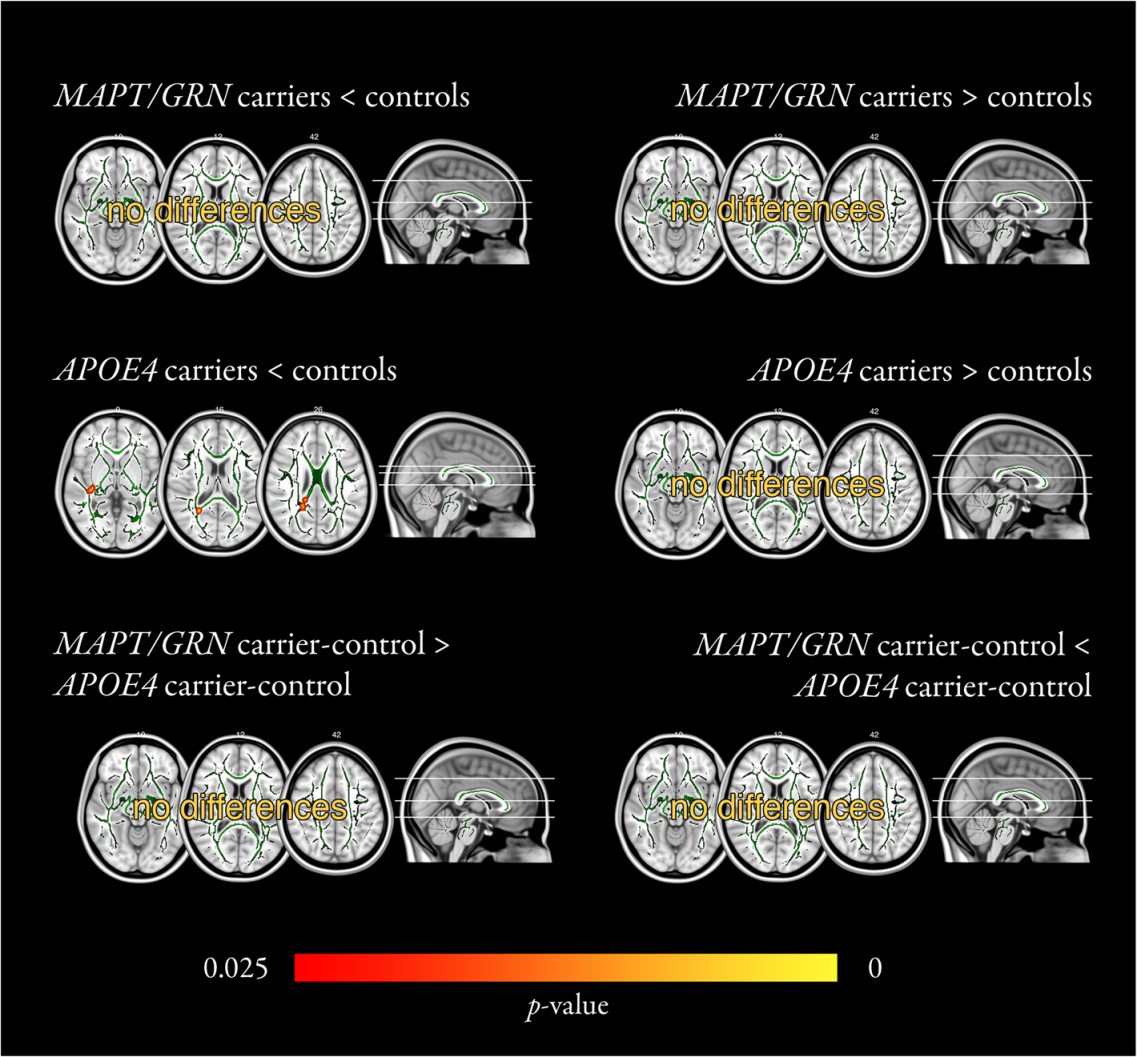
White matter FA analysis. Differences in FA (or lack thereof) are shown for each contrast (e.g., *MAPT/GRN* mutation carriers greater or smaller than controls; *APOE4* carriers greater or smaller than controls; *MAPT/GRN* carrier-control differences greater or smaller than *APOE4* carrier-control differences). Mean skeleton maps are shown in green; skeletonised significant results were thickened for better visualisation. Four clusters of FA reductions were found in *APOE4* carriers compared to controls (middle left panel). Colour bar represents significance. *APOE4, apolipoprotein E ε4*; FA, fractional anisotropy; *MAPT/GRN*, *microtubule-associated protein tau / progranulin*

Tract-based spatial statistics revealed no FA, MD, AxD, or RD differences between *MAPT/GRN* mutation carriers and controls. However, we found four clusters of FA reductions in *APOE4* carriers compared to controls (Fig. 2, Table 2). Three clusters were located in the forceps major, more specifically in right side of the callosal splenium, and one cluster was located in the right inferior fronto-occipital fasciculus. We found no significant differences between the *MAPT/GRN* and *APOE4* carrier-control contrasts in our whole-brain analysis, nor in our region-of-interest analyses.

**Table 2:** Cluster information

Cluster	Size	Max *t*-statistic	MNI coordinates			L/R	Area (peak voxel)
			x	y	z		
1	64	4.14	54	101	72	R	IFOF
2	44	3.19	71	79	95	R	Splenium
3	32	3.58	63	73	87	R	Splenium
4	22	4.19	74	87	98	R	Splenium

Cluster information for significant clusters of reduced FA in *APOE4* carriers compared to controls. Minimum cluster size was 10.

*APOE4, Apolipoprotein E ε4* carriers; IFOF, Inferior fronto-occipital fasciculus

### Functional connectivity

We found no differences in region-to-network functional connectivity in *MAPT/GRN* mutation carriers compared to controls, in *APOE4* carriers compared to controls, or between the two carrier-control contrasts in any of the five resting-state networks.

### Heterogeneity analyses

Analyses including covariates for the difference between *MAPT* and *GRN* mutations, and between *APOE4* hetero- and homozygosity yielded similar results as our main analyses. There were no grey matter volume differences between *MAPT/GRN* mutation carriers and controls, *APOE4* carriers and controls, or between the two carrier-control contrasts. *APOE4* carriers had reduced FA in compared to controls (Additional file 2: Figure S2), though only one of the four clusters remained significant. We found no DTI differences between *MAPT/GRN* mutation carriers and controls, nor between the two carrier-control contrasts. We found no differences in region-to-network functional connectivity in *MAPT/GRN* mutation carriers compared to controls, *APOE4* carriers compared to controls, or between the carrier-control contrasts in any of the five resting-state networks.

### Data availability

All non-thresholded statistical images for grey matter volume, white matter diffusion, and functional connectivity results of our default analysis can be found on NeuroVault [74]: https://neurovault.org/collections/NXLXKVCZ/.

## Discussion

Differences in atrophy, white matter diffusion, and functional connectivity patterns have been repeatedly shown between FTD and AD patients [11–16], and between asymptomatic mutation carriers at risk for these diseases and controls (e.g., *MAPT* and *GRN* mutation carriers [29–33]; *APOE4* carriers [34–43]). However, comparisons between groups at risk for FTD and groups at risk for AD have been lacking, even though early-stage differences between these dementias are key to improve on diagnostic standards. In this study, we aimed to replicate previously found differences in asymptomatic mutation carriers at risk for FTD and AD compared to their respective control groups. More importantly, we investigated whether carrier-control differences diverged, similar to the divergences that exist between FTD and AD. While we could replicate some of the previously reported fractional anisotropy reductions in asymptomatic *APOE4* carriers, we found no evidence of divergence between *MAPT/GRN* carrier-control differences and *APOE4* carrier-control differences, even when restricting our DTI analysis to regions which are known to differ between FTD and AD patients. This may suggest that the neuroimaging biomarkers measured in this study are not sufficiently specific to differentiate between FTD-related pathology and pathology possibly related to AD at this early stage.

Our lack of differences between groups in grey matter volume were unsurprising. In asymptomatic risk mutation carriers, one would not expect dementia related atrophy unless the carrier would be close to symptom onset. Indeed, grey matter volume differences have not been reported in asymptomatic *MAPT/GRN* mutation carriers [31, 32], though reports in asymptomatic *APOE4* carriers have been conflicting. While some groups report no grey matter volume differences in asymptomatic *APOE4* carriers [35, 39, 40, 43], others found reduced grey matter volume in the hippocampus [36, 75], lingual gyrus [36], precuneus [36, 76], insula [76], caudate nucleus, precentral gyrus, and cerebellar crus [75]. These conflicting findings may in part result from methodological differences, sample sizes, and the different age ranges between studies. Since disease modifying treatments aim to prevent atrophy, one would ideally aim to diagnose dementia patients before atrophy occurs to maximise potential treatment effect. Accordingly, biomarker research should focus on detecting substrates of neurodegeneration that precede atrophy and that may be reversible by future disease modifying treatments.

White matter diffusion analyses yielded areas of reduced FA in *APOE4* carriers compared to controls in the splenium of the corpus callosum, and in the right inferior fronto-occipital fasciculus. These results concur with previous reports in *APOE4* carriers. FA reductions were most often reported in the corpus callosum, cingulum, and inferior fronto-occipital fasciculi [39, 44–51], while FA differences in the corticospinal tract [39, 49–51] and superior longitudinal fasciculi [39, 50, 51] were less frequently reported. We found no diffusion differences in *MAPT/GRN* mutation carriers compared to controls, in contrast to earlier work [32, 33]. However, this might be explained by differences in methodology. One study found significant FA reductions only within certain pre-specified tracts, and, similar to our current study, found no whole-brain differences [32]. The other study found differences at *p*<0.005 uncorrected for multiple comparisons across space. Our analyses were performed with a more restrictive significance level, as we corrected for multiple comparisons across space using threshold-free cluster enhancement, and used the statistical threshold appropriate for a two-sided test, which is not a standard procedure in neuroimaging [77]. Interestingly, DTI alterations are larger in FTD patients than in AD patients [11, 13–16], while preclinical alterations in *APOE4* carriers [39, 44–51] are more widespread than in *MAPT/GRN* mutation carriers [32, 33]. Recently, it has been postulated that white matter DTI differences in genetic FTD develop rather explosively in the years just prior to symptom onset [78, 79]. This might explain why in our sample, we found DTI differences in *APOE4* carriers, but no DTI differences in *MAPT/GRN* mutation carriers. Although there were FA reductions in *APOE4* carriers compared to controls, the difference was not strong enough to result in a difference between the *MAPT/GRN* carrier-control contrast and the *APOE4* carrier-control contrast. We also performed region-of-interest analyses in the uncinate fasciculi, forceps minor, or bilateral anterior thalamic radiations, which were found to have FA reductions and RD increases in FTD patients compared to AD patients [11, 13–16]. However, even in these regions of interest, we could not find DTI differences between the *MAPT/GRN* carrier-control contrast and the *APOE4* carrier-control contrast. As such, we could not conclude that *MAPT/GRN* mutation carriership had a different effect on white matter diffusion metrics than *APOE4* carriership.

It has been previously argued that the default mode network and the salience network are inversely correlated and both play a role in AD and FTD. Specifically, functional connectivity in the default mode network was reported to be reduced in AD patients and increased in FTD patients, whereas functional connectivity in the salience network was reported to be inversely affected: reduced in FTD patients and increased in AD patients [12, 17]. In asymptomatic *APOE4* carriers, this inverse correlation was also shown. Functional connectivity with the default mode network was decreased and functional connectivity with the salience network was enhanced in *APOE4* carriers compared to controls [41]. In asymptomatic *MAPT* and *GRN* mutation carriers, functional connectivity was reduced in the salience network, but no differences in the default mode network were found [32]. Based on these results, we hypothesised that functional connectivity in the default mode network and salience network would be ideal candidates to screen for early changes in asymptomatic risk carriers. However, we found no evidence of functional connectivity differences, either between the respective carrier and control groups or divergent differences between the carrier-control contrasts. This might in part be a power issue but could also be explained by population and methodological differences. For example, our sample was on average younger and had a broader age range than the *APOE4* sample investigated by Machulda et al. (2011) [41]. Furthermore, we performed data-driven dual regression analyses, whereas both Machulda et al. (2011) [41] and Dopper et al. (2014) [32] performed seed-based analyses. While small seed areas are arbitrarily placed and may be subject to registration mismatch, dual regression networks are less sensitive to these issues due to their data-driven origin. Indeed, dual regression is amongst the best functional MRI analysis techniques in terms of test-retest reliability [80, 81]. Therefore, the most likely explanation of our functional connectivity results is that our groups were on average too far from symptom onset for functional connectivity alterations in the default mode network and salience network to robustly appear.

Strengths of this study include its unique design to pick up differences between FTD- and AD-related pathology in asymptomatic populations, and the inclusion of control groups from both sites to deal with potential scan site bias. We performed specific region-of-interest analyses to increase power to find differences in DTI metrics. Furthermore, we used FIX [62, 63] to clean up structured noise (e.g., motion, artefacts) from rs-fMRI data to reduce scanner-based functional connectivity differences [64] and increase the signal-to-noise ratio. To account for possible heterogeneity resulting from pooling *MAPT* and *GRN* mutation carriers, and *APOE4* hetero- and homozygotes, we performed additional analyses including covariates for the different mutation types. The results of these analyses were very similar to our main results, suggesting that the effect of genetic heterogeneity in our main analyses was altogether limited. Limitations must also be considered. Firstly, differences in penetrance and age of onset exist between *MAPT/GRN* and *APOE4*. *MAPT* and *GRN* mutations have an autosomal dominant inheritance pattern, and are highly penetrant [82, 83]. On the other hand, *APOE4* has a dose-dependent effect on lifetime AD risk. Heterozygous *APOE4* carriers have an estimated lifetime risk for AD of approximately 25%, while *APOE4* homozygosity is associated with an estimated lifetime risk of around 55% [84]. Therefore, it is unlikely that all *APOE4* carriers from our sample will develop AD, which reduced our power to detect AD-related differences. For the same reason, it cannot be entirely ruled out that some of the differences associated with the *APOE4* carriers do not reflect presymptomatic AD-related pathology. Information on *MAPT/GRN* mutation carriership was not available for *APOE4* carriers and controls, and information on *APOE4* carriership was not available for *MAPT/GRN* mutation carriers and controls. Due to the infrequency of *MAPT* and *GRN* mutations, it is unlikely that *APOE4* carriers or controls had an *MAPT* or *GRN* mutation. However, the frequency of the *APOE4* allele in Caucasian populations is around 14% [85], and it is likely that some of the *MAPT* and *GRN* mutation carriers and controls had an *APOE4* allele. As *MAPT/GRN* mutation carriers and controls were from the same families, the frequency of the *APOE4* alleles within these groups was most likely similar. Therefore, the effect of *APOE4* on our *MAPT/GRN* analyses is presumably small. The broad age range in our groups presents another limitation. FTD- or AD-related pathology may be absent or present in a lesser degree in young mutation carriers than in older carriers, who are closer to symptom-onset. However, even though a broad age range was present in our sample, physiological brain aging effects are unlikely to have influenced our results. The four groups were matched for age, and age was added as confound covariate to the model. Therefore, physiological brain aging effects should be equally distributed across groups and were accounted for in the model. In order to increase power, future neuroimaging research comparing FTD- and AD-related pathology in asymptomatic risk groups should contain clinical follow-up and conversion information, which will enable the inclusion of a time to onset variable to the model.

Dementias are relentlessly progressive diseases for which no adequate treatments currently exist, and differentiation between various forms of dementia is clinically challenging. Recently, MRI has shown different patterns of grey matter atrophy, DTI alterations and functional connectivity differences in AD and FTD patients [11–17]. However, early differential identification of at-risk groups is key to study pathophysiological processes, develop disease-modulating drugs and, eventually, identify patient groups that may benefit from these treatments. In the current study, we could not find differences suggestive of divergent pathways of underlying FTD and AD pathology in asymptomatic risk mutation carriers.

## Supplementary information

**Additional file 1: Figure S1.** Dual regression. Subject-specific spatial maps for statistical testing are acquired from group-level ICA spatial maps in two steps. First, group-level ICA spatial maps are used as spatial regressor on each subject’s rs-fMRI data to obtain time series associated with those ICA components (Step 1). Next, these time series are used as temporal regressor to obtain subject-specific spatial maps for each component (Step 2). These maps are then used for voxel-wise statistical testing. GICA, group-level independent component analysis; rs-fMRI, resting-state functional magnetic resonance imaging.

**Additional file 2: Figure S2.** White matter FA analysis with mutation covariates. In this analysis, covariates were added for the difference between *MAPT* and *GRN* mutations, and between *APOE4* hetero- and homozygosity to account for genetic heterogeneity. Differences in FA (or lack thereof) are shown for each contrast (e.g., *MAPT/GRN* mutation carriers greater or smaller than controls; *APOE4* carriers greater or smaller than controls; *MAPT/GRN* carrier-control differences greater or smaller than *APOE4* carrier-control differences). Mean skeleton maps are shown in green; skeletonised significant results were thickened for better visualisation. One cluster of FA reductions was found in *APOE4* carriers compared to controls (middle left panel). Colour bar represents significance. *APOE4, apolipoprotein E ε4*; FA, fractional anisotropy; *MAPT/GRN*, *microtubule-associated protein tau / progranulin*.

## Data Availability

The datasets and scripts used during the current study are available from the corresponding author on reasonable request. All non-thresholded statistical images for grey matter volume, white matter diffusion, and functional connectivity results can be found on NeuroVault [74]: https://neurovault.org/collections/NXLXKVCZ/.

## Abbreviations

(rs-f)MRI: (resting-state-functional) Magnetic resonance imaging
AD: Alzheimer’s disease
*APOE4*: *Apolipoprotein E ε4*
AxD: Axial diffusivity
DTI: Diffusion tensor imaging
FA: Fractional anisotropy
FIX: FMRIB’s ICA-based X-noiseifier
FMRIB: Functional Magnetic Resonance Imaging of the Brain Centre
FSL: FMRIB Software Library
FTD: Frontotemporal dementia
*GRN*: *Progranulin*
ICA: Independent component analysis
LUMC: Leiden University Medical Centre
MAPT: Microtubule-associated protein Tau
MD: Mean diffusivity
RD: Radial diffusivity

## References

[1] Lobo A, Launer LJ, Fratiglioni L (2000). Prevalence of dementia and major subtypes in Europe: A collaborative study of population-based cohorts. Neurology.

[2] Plassman BL, Langa KM, Fisher GG (2007). Prevalence of dementia in the United States: The aging, demographics, and memory study. Neuroepidemiology.

[3] Seelaar H, Kamphorst W, Rosso SM (2008). Distinct genetic forms of frontotemporal dementia. Neurology..

[4] Vieira RT, Caixeta L, Machado S (2013). Epidemiology of early-onset dementia: a review of the literature. Clin Pract Epidemiol Ment Health.

[5] Rascovsky K, Hodges JR, Knopman D (2011). Sensitivity of revised diagnostic criteria for the behavioural variant of frontotemporal dementia. Brain.

[6] Gorno-Tempini ML, Hillis AE, Weintraub S (2011). Classification of primary progressive aphasia and its variants. Neurology.

[7] McKhann GM, Knopman DS, Chertkow H (2011). The diagnosis of dementia due to Alzheimer’s disease: Recommendations from the National Institute on Aging-Alzheimer’s Association workgroups on diagnostic guidelines for Alzheimer’s disease. Alzheimers Dement.

[8] Seelaar H, Rohrer JD, Pijnenburg YAL, Fox NC, van Swieten JC (2011). Clinical, genetic and pathological heterogeneity of frontotemporal dementia: a review. J Neurol Neurosurg Psychiatry.

[9] Galimberti D, Scarpini E (2012). Clinical phenotypes and genetic biomarkers of FTLD. J Neural Transm.

[10] Seeley WW, Allman JM, Carlin DA (2007). Divergent social functioning in behavioral variant frontotemporal dementia and Alzheimer disease: reciprocal networks and neuronal evolution. Alzheimer Dis Assoc Disord.

[11] Zhang Y, Schuff N, Du A-T (2009). White matter damage in frontotemporal dementia and Alzheimer’s disease measured by diffusion MRI. Brain.

[12] Zhou J, Greicius MD, Gennatas ED (2010). Divergent network connectivity changes in behavioural variant frontotemporal dementia and Alzheimer’s disease. Brain.

[13] Zhang Y, Schuff N, Ching C, et al. Joint assessment of structural, perfusion, and diffusion MRI in Alzheimer’s disease and frontotemporal dementia. Int J Alzheimers Dis. 2011;546871. 10.4061/2011/546871 PMC3132541 21760989

[14] Mahoney CJ, Ridgway GR, Malone IB (2014). Profiles of white matter tract pathology in frontotemporal dementia. Hum Brain Mapp.

[15] Möller C, Hafkemeijer A, Pijnenburg YAL (2015). Joint assessment of white matter integrity, cortical and subcortical atrophy to distinguish AD from behavioral variant FTD: A two-center study. NeuroImage Clin.

[16] Daianu M, Mendez MF, Baboyan VG (2016). An advanced white matter tract analysis in frontotemporal dementia and early-onset Alzheimer’s disease. Brain Imaging Behav.

[17] Tuovinen T, Rytty R, Moilanen V (2017). The Effect of Gray Matter ICA and Coefficient of Variation Mapping of BOLD Data on the Detection of Functional Connectivity Changes in Alzheimer’s Disease and bvFTD. Front Hum Neurosci.

[18] Graham A, Davies R, Xuereb J (2005). Pathologically proven frontotemporal dementia presenting with severe amnesia. Brain.

[19] Le Ber I, Camuzat A, Hannequin D (2008). Phenotype variability in progranulin mutation carriers: a clinical, neuropsychological, imaging and genetic study. Brain.

[20] Johnson Julene K., Head Elizabeth, Kim Ronald, Starr Arnold, Cotman Carl W. (1999). Clinical and Pathological Evidence for a Frontal Variant of Alzheimer Disease. Archives of Neurology.

[21] Mendez Mario F., Shapira Jill S., McMurtray Aaron, Licht Eliot, Miller Bruce L. (2007). Accuracy of the Clinical Evaluation for Frontotemporal Dementia. Archives of Neurology.

[22] Forman MS, Farmer J, Johnson JK (2006). Frontotemporal dementia: Clinicopathological correlations. Ann Neurol.

[23] Knibb JA, Xuereb JH, Patterson K, Hodges JR (2006). Clinical and pathological characterization of progressive aphasia. Ann Neurol.

[24] Alladi S, Xuereb J, Bak T (2007). Focal cortical presentations of Alzheimer’s disease. Brain.

[25] Mohs RC, Doody RS, Morris JC (2001). A 1-year, placebo-controlled preservation of function survival study of donepezil in AD patients. Neurology.

[26] Mendez MF, Giannakopoulos P, Hof PR (2009). Frontotemporal Dementia: Therapeutic Interventions. Dementia in Clinical Practice.

[27] Pressman PS, Miller BL (2014). Diagnosis and Management of Behavioral Variant Frontotemporal Dementia. Biol Psychiatry.

[28] Rabinovici GD, Miller BL (2010). Frontotemporal Lobar Degeneration. CNS Drugs.

[29] Whitwell JL, Weigand SD, Gunter JL (2011). Trajectories of brain and hippocampal atrophy in FTD with mutations in MAPT or GRN. Neurology.

[30] Whitwell JL, Josephs KA, Avula R (2011). Altered functional connectivity in asymptomatic MAPT subjects: a comparison to bvFTD. Neurology.

[31] Borroni B, Alberici A, Cercignani M (2012). Granulin mutation drives brain damage and reorganization from preclinical to symptomatic FTLD. Neurobiol Aging.

[32] Dopper EGP, Rombouts SARB, Jiskoot LC (2014). Structural and functional brain connectivity in presymptomatic familial frontotemporal dementia. Neurology.

[33] Pievani M, Paternicò D, Benussi L (2014). Pattern of structural and functional brain abnormalities in asymptomatic granulin mutation carriers. Alzheimers Dement.

[34] Nierenberg J, Pomara N, Hoptman MJ, Sidtis JJ, Ardekani BA, Lim KO (2005). Abnormal white matter integrity in healthy apolipoprotein E epsilon4 carriers. Neuroreport.

[35] Cherbuin N, Anstey KJ, Sachdev PS (2008). Total and regional gray matter volume is not related to APOE*E4 status in a community sample of middle-aged individuals. J Gerontol Ser A.

[36] Honea RA, Vidoni E, Harsha A, Burns JM (2009). Impact of APOE on the Healthy Aging Brain: A Voxel-Based MRI and DTI Study. J Alzheimers Dis.

[37] Filippini N, MacIntosh BJ, Hough MG (2009). Distinct patterns of brain activity in young carriers of the APOE-epsilon4 allele. Proc Natl Acad Sci U S A.

[38] Agosta F, Vossel KA, Miller BL (2009). Apolipoprotein E epsilon4 is associated with disease-specific effects on brain atrophy in Alzheimer’s disease and frontotemporal dementia. Proc Natl Acad Sci U S A.

[39] Heise V, Filippini N, Ebmeier KP, Mackay CE (2011). The APOE ɛ4 allele modulates brain white matter integrity in healthy adults. Mol Psychiatry.

[40] Filippini N, Ebmeier KP, MacIntosh BJ (2011). Differential effects of the APOE genotype on brain function across the lifespan. Neuroimage.

[41] Machulda MM, Jones DT, Vemuri P, et al. Effect of APOE ε4 Status on Intrinsic Network Connectivity in Cognitively Normal Elderly Subjects. Arch Neurol. 2011;68(9):1131–6. 10.1001/archneurol.2011.108 PMC3392960 21555604

[42] Trachtenberg AJ, Filippini N, Ebmeier KP, Smith SM, Karpe F, Mackay CE (2012). The effects of APOE on the functional architecture of the resting brain. Neuroimage.

[43] Matura S, Prvulovic D, Jurcoane A (2014). Differential effects of the ApoE4 genotype on brain structure and function. Neuroimage.

[44] Persson J, Lind J, Larsson A (2006). Altered brain white matter integrity in healthy carriers of the APOE epsilon4 allele: a risk for AD?. Neurology.

[45] Smith CD, Chebrolu H, Andersen AH (2010). White matter diffusion alterations in normal women at risk of Alzheimer’s disease. Neurobiol Aging.

[46] Gold BT, Powell DK, Andersen AH, Smith CD (2010). Alterations in multiple measures of white matter integrity in normal women at high risk for Alzheimer’s disease. Neuroimage.

[47] Adluru N, Destiche DJ, Lu SY-F (2014). White matter microstructure in late middle-age: Effects of apolipoprotein E4 and parental family history of Alzheimer’s disease. NeuroImage Clin.

[48] Lyall DM, Harris SE, Bastin ME (2014). Alzheimer’s disease susceptibility genes APOE and TOMM40, and brain white matter integrity in the Lothian Birth Cohort 1936. Neurobiol Aging.

[49] Laukka Erika J., Lövdén Martin, Kalpouzos Grégoria, Papenberg Goran, Keller Lina, Graff Caroline, Li Tie-Qiang, Fratiglioni Laura, Bäckman Lars (2015). Microstructural White Matter Properties Mediate the Association between APOE and Perceptual Speed in Very Old Persons without Dementia. PLOS ONE.

[50] Cavedo E, Lista S, Rojkova K (2017). Disrupted white matter structural networks in healthy older adult APOE ε4 carriers – An international multicenter DTI study. Neuroscience.

[51] Operto G, Cacciaglia R, Grau-Rivera O (2018). White matter microstructure is altered in cognitively normal middle-aged APOE-ε4 homozygotes. Alzheimers Res Ther.

[52] Ludolph A, Drory V, Hardiman O (2015). A revision of the El Escorial criteria - 2015. Amyotroph Lateral Scler Front Degener.

[53] Jenkinson M, Beckmann CF, Behrens TEJ, Woolrich MW, Smith SM (2012). FSL. Neuroimage.

[54] Douaud G, Smith SM, Jenkinson M (2007). Anatomically related grey and white matter abnormalities in adolescent-onset schizophrenia. Brain.

[55] Good CD, Johnsrude IS, Ashburner J, Henson RNA, Friston KJ, Frackowiak RSJ (2001). A Voxel-Based Morphometric Study of Ageing in 465 Normal Adult Human Brains. Neuroimage.

[56] Smith SM, Jenkinson M, Woolrich MW (2004). Advances in functional and structural MR image analysis and implementation as FSL. Neuroimage..

[57] Anderson JLR, Jenkinson M, Smith SM (2007). Non-linear registration aka Spatial normalisation. FMRIB Technical Report TR07JA2 [Internet].

[58] Smith SM, Jenkinson M, Johansen-Berg H (2006). Tract-based spatial statistics: Voxelwise analysis of multi-subject diffusion data. Neuroimage.

[59] Jenkinson M, Bannister P, Brady M, Smith SM (2002). Improved optimization for the robust and accurate linear registration and motion correction of brain images. Neuroimage.

[60] Jenkinson M, Smith SM (2001). A global optimisation method for robust affine registration of brain images. Med Image Anal.

[61] Greve DN, Fischl B (2009). Accurate and Robust Brain Image Alignment using Boundary-based Registration. Neuroimage.

[62] Griffanti L, Salimi-Khorshidi G, Beckmann CF (2014). ICA-based artefact removal and accelerated fMRI acquisition for improved resting state network imaging. Neuroimage.

[63] Salimi-Khorshidi G, Douaud G, Beckmann CF, Glasser MF, Griffanti L, Smith SM (2014). Automatic denoising of functional MRI data: Combining independent component analysis and hierarchical fusion of classifiers. Neuroimage.

[64] Feis RA, Smith SM, Filippini N (2015). ICA-based artifact removal diminishes scan site differences in multi-center resting-state fMRI. Front Neurosci.

[65] Hyvärinen A (1999). Fast and robust fixed-point algorithms for independent component analysis. IEEE Trans Neural Netw.

[66] Beckmann CF, Smith SM (2004). Probabilistic independent component analysis for functional magnetic resonance imaging. IEEE Trans Med Imaging.

[67] Beckmann CF, DeLuca M, Devlin JT, Smith SM (2005). Investigations into resting-state connectivity using independent component analysis. Philos Trans R Soc B Biol Sci.

[68] Damoiseaux JS, Rombouts SARB, Barkhof F (2006). Consistent resting-state networks across healthy subjects. Proc Natl Acad Sci U S A.

[69] Rytty R, Nikkinen J, Paavola L (2013). GroupICA dual regression analysis of resting state networks in a behavioral variant of frontotemporal dementia. Front Hum Neurosci.

[70] Tian L, Kong Y, Ren J, Varoquaux G, Zang Y-F, Smith SM (2013). Spatial vs. Temporal Features in ICA of Resting-State fMRI - A Quantitative and Qualitative Investigation in the Context of Response Inhibition. PLoS One.

[71] Bey K, Montag C, Reuter M, Weber B, Markett S (2015). Susceptibility to everyday cognitive failure is reflected in functional network interactions in the resting brain. Neuroimage.

[72] Beckmann CF, Mackay CE, Filippini N, Smith SM. Group comparison of resting-state FMRI data using multi-subject ICA and dual regression: OHBM; 2009.

[73] Winkler AM, Ridgway GR, Webster MA, Smith SM, Nichols TE (2014). Permutation inference for the general linear model. Neuroimage.

[74] Gorgolewski KJ, Varoquaux G, Rivera G (2015). NeuroVault.org: a web-based repository for collecting and sharing unthresholded statistical maps of the human brain. Front Neuroinform.

[75] Cacciaglia R, Molinuevo JL, Falcón C (2018). Effects of APOE-ε4 allele load on brain morphology in a cohort of middle-aged healthy individuals with enriched genetic risk for Alzheimer’s disease. Alzheimers Dement.

[76] ten Kate M, Sanz-Arigita EJ, Tijms BM (2016). Impact of APOE-ɛ4 and family history of dementia on gray matter atrophy in cognitively healthy middle-aged adults. Neurobiol Aging.

[77] Chen G, Cox RW, Glen DR, Rajendra JK, Reynolds RC, Taylor PA (2019). A tail of two sides: Artificially doubled false positive rates in neuroimaging due to the sidedness choice with t-tests. Hum Brain Mapp.

[78] Jiskoot LC, Panman JL, Meeter LHH (2019). Longitudinal multimodal MRI as prognostic and diagnostic biomarker in presymptomatic familial frontotemporal dementia. Brain.

[79] Feis RA, Bouts MJRJ, de Vos F (2019). A multimodal MRI-based classification signature emerges just prior to symptom onset in frontotemporal dementia mutation carriers. J Neurol Neurosurg Psychiatry.

[80] Zuo X-N, Kelly C, Adelstein JS, Klein DF, Castellanos FX, Milham MP (2010). Reliable intrinsic connectivity networks: Test–retest evaluation using ICA and dual regression approach. Neuroimage.

[81] Zuo X-N, Xing X-X (2014). Test-retest reliabilities of resting-state FMRI measurements in human brain functional connectomics: A systems neuroscience perspective. Neurosci Biobehav Rev.

[82] van Swieten JC, Rosso SM, Heutink P, Adam MP, Ardinger HH, Pagon RA (2000). MAPT-Related Disorders. GeneReviews®.

[83] van Swieten JC, Heutink P (2008). Mutations in progranulin (GRN) within the spectrum of clinical and pathological phenotypes of frontotemporal dementia. Lancet Neurol.

[84] Genin E, Hannequin D, Wallon D (2011). APOE and Alzheimer disease: a major gene with semi-dominant inheritance. Mol Psychiatry.

[85] Eisenberg DTA, Kuzawa CW, Hayes MG (2010). Worldwide allele frequencies of the human apolipoprotein E gene: Climate, local adaptations, and evolutionary history. Am J Phys Anthropol.

