# Supplementary figures and images for: Multimodal MRI of grey matter, white matter, and functional connectivity in cognitively healthy mutation carriers at risk for frontotemporal dementia and Alzheimer's disease
Source: BMC Neurol. 2019 Dec 27;19:343. doi: 10.1186/s12883-019-1567-0 (PMC6933911; doi:10.1186/s12883-019-1567-0)

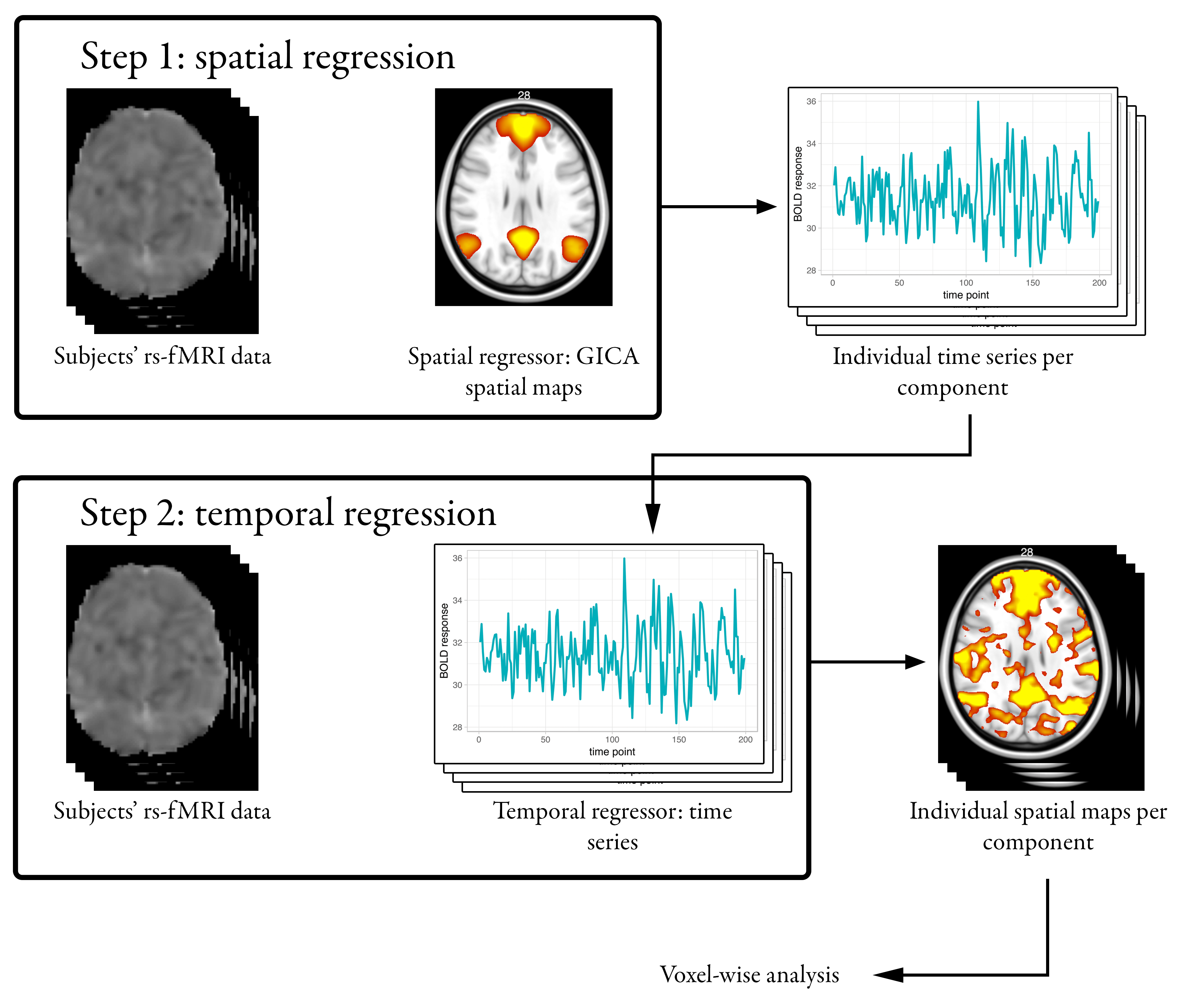

Supplement: Supplementary file 1 — Additional file 1: Figure S1. Dual regression. Subject-specific spatial maps for statistical testing are acquired from group-level ICA spatial maps in two steps. First, group-level ICA spatial maps are used as spatial regressor on each subject’s rs-fMRI data to obtain time series associated with those ICA components (Step 1). Next, these time series are used as temporal regressor to obtain subject-specific spatial maps for each component (Step 2). These maps are then used for voxel-wise statistical testing. GICA, group-level independent component analysis; rs-fMRI, resting-state functional magnetic resonance imaging. [file 12883_2019_1567_MOESM1_ESM.tif]

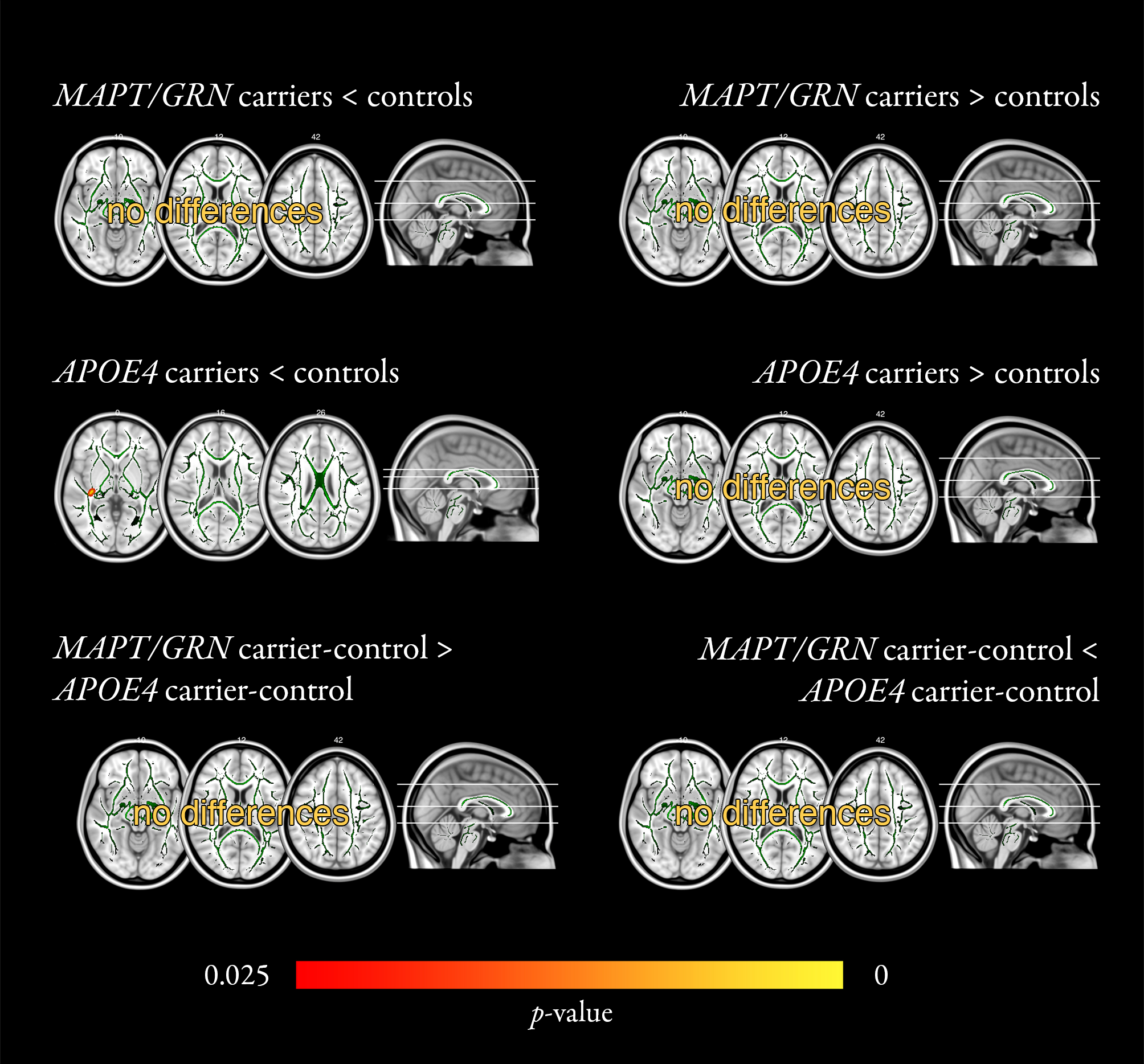

Supplement: Supplementary file 2 — Additional file 2: Figure S2. White matter FA analysis with mutation covariates. In this analysis, covariates were added for the difference between MAPT and GRN mutations, and between APOE4 hetero- and homozygosity to account for genetic heterogeneity. Differences in FA (or lack thereof) are shown for each contrast (e.g., MAPT/GRN mutation carriers greater or smaller than controls; APOE4 carriers greater or smaller than controls; MAPT/GRN carrier-control differences greater or smaller than APOE4 carrier-control differences). Mean skeleton maps are shown in green; skeletonised significant results were thickened for better visualisation. One cluster of FA reductions was found in APOE4 carriers compared to controls (middle left panel). Colour bar represents significance. APOE4, apolipoprotein E ε4; FA, fractional anisotropy; MAPT/GRN, microtubule-associated protein tau / progranulin. [file 12883_2019_1567_MOESM2_ESM.tif]
